# Myoelectric interface for neurorehabilitation conditioning to reduce abnormal leg co-activation after stroke: a pilot study

**DOI:** 10.1186/s12984-024-01305-0

**Published:** 2024-01-20

**Authors:** Abed Khorasani, Joel Hulsizer, Vivek Paul, Cynthia Gorski, Yasin Y. Dhaher, Marc W. Slutzky

**Affiliations:** 1 Department of Neurology, Northwestern University, 320 East Superior Ave., Searle 11-473, 60611 Chicago, IL USA; 2 Department of Physical Medicine & Rehabilitation, Northwestern University, Chicago, IL USA; 3 Department of Neuroscience, Northwestern University, Chicago, IL USA; 4 Department of Biomedical Engineering, Northwestern University, Evanston, IL USA; 5 Peter O’Donnell Jr. Brain Institute, University of Texas Southwestern Medical Center, Dallas, TX USA; 6 Department of Physical Medicine and Rehabilitation, University of Texas Southwestern Medical Center, Dallas, TX USA

**Keywords:** Stroke, Gait, Co-activation, EMG, Game-based rehabilitation, Knee flexion

## Abstract

**Background:**

The ability to walk is an important factor in quality of life after stroke. Co-activation of hip adductors and knee extensors has been shown to correlate with gait impairment. We have shown previously that training with a myoelectric interface for neurorehabilitation (MINT) can reduce abnormal muscle co-activation in the arms of stroke survivors.

**Methods:**

Here, we extend MINT conditioning to stroke survivors with leg impairment. The aim of this pilot study was to assess the safety and feasibility of using MINT to reduce abnormal co-activation between hip adductors and knee extensors and assess any effects on gait. Nine stroke survivors with moderate to severe gait impairment received 6 h of MINT conditioning over six sessions, either in the laboratory or at home.

**Results:**

MINT participants completed a mean of 159 repetitions per session without any adverse events. Further, participants learned to isolate their muscles effectively, resulting in a mean reduction of co-activation of 70% compared to baseline. Moreover, gait speed increased by a mean of 0.15 m/s, more than the minimum clinically important difference. Knee flexion angle increased substantially, and hip circumduction decreased.

**Conclusion:**

MINT conditioning is safe, feasible at home, and enables reduction of co-activation in the leg. Further investigation of MINT’s potential to improve leg movement and function after stroke is warranted. Abnormal co-activation of hip adductors and knee extensors may contribute to impaired gait after stroke.

*Trial registration* This study was registered at ClinicalTrials.gov (NCT03401762, Registered 15 January 2018, https://clinicaltrials.gov/study/NCT03401762?tab=history&a=4).

## Background

Impaired lower limb function following stroke results in impaired walking and an increased risk of falling [1]. While some stroke survivors achieve independent walking status, about a third continue to face challenges related to lower limb coordination, gait speed, walking endurance, and balance [2, 3]. Impaired movement after a stroke is often caused by a combination of weakness, spasticity, and abnormal muscle co-activation [4–7]. While many rehabilitation approaches have been developed to address weakness and spasticity, gait dysfunction can remain severe despite reduction of these components [8–10]. This suggests that abnormal muscle co-activation is a crucial contributor to gait dysfunction in many hemiparetic stroke survivors.

Stroke survivors often exhibit abnormal gait kinematics, including abnormal pelvic and leg joint motion in both the sagittal (decreased knee flexion) and frontal planes (hip hiking, circumduction) [11–13]. These abnormal kinematics are mechanically inefficient and energetically costly, which increases fatigue [14–16]. There is some evidence that hip hiking and circumduction are compensatory mechanisms to ensure toe clearance in people with stiff-knee gait [17, 18]. However, when external assistance to knee flexion was applied to hemiparetic legs using an orthosis, no changes in the expression of hip hiking and circumduction was observed [13]. Further, neurotypical participants whose knee flexion was artificially restricted with an orthosis did not show compensatory circumduction [19]. These findings suggest that the abnormal kinematics may be the result of compensating for an abnormal coupling between hip adduction and knee extension, instead of compensating for reduced knee flexion [12, 20]. An increase in knee extension and hip adduction at or near toe-off reduces the minimum distance between the toe and the ground, and between the foot and the contralateral leg, respectively, thus increasing the risk of tripping. To clear the ground and avoid hitting the opposite leg, patients may hip hike and circumduct [21]. While multiple abnormal coactivation patterns are seen after stroke [22–25], abnormal hip adduction/knee extension, especially at toe-off, was the dominant pattern [12]*.* Further, in a multiple regression model incorporating both classical impairments (decreased flexion of hip, knee, or ankle) and abnormal hip/knee coupling, abnormal hip adduction/knee extension most strongly correlated with hip hiking and most strongly predicted overground walking speed [22]. These studies were largely correlational. We have developed a system to reduce co-activation, called a myoelectric interface for neurorehabilitation (MINT), and shown that it effectively reduces co-activation between arm muscles trained [26, 27] and may improve arm function [28, 29].

Here, we tested the hypothesis that reducing abnormal hip adduction/knee extension co-activation in leg muscles through MINT conditioning could lead to improved walking function and joint biomechanics. We investigated this in nine stroke survivors in the lab and at home.

## Methods

### Participants and EMG recording

This study was conducted in accordance with the STROBE (Strengthening the Reporting of Observational Studies in Epidemiology) cohort reporting guidelines, ensuring the comprehensive and transparent reporting of key elements and essential findings [30]. The study was conducted with approval from the Institutional Review Board of Northwestern University as part of a larger study investigating MINT for improving arm function (NCT03401762), and all participants provided written informed consent before eligibility assessment. We enrolled 9 adult chronic stroke survivors in Chicago, Illinois from September 2021 to February 2023. These participants had experienced a first-time stroke at least 6 months prior and had moderate to severe gait impairment. We excluded individuals who had impairments in vision, memory, language, or concentration, received botulinum toxin on the affected leg within the previous 3 months, or were currently participating in another research study involving the leg. The small sample size of nine participants was chosen for our pilot clinical study to focus on evaluating the initial safety and feasibility of MINT training for the leg, both in the lab and at home, in chronic patients with moderate to severe walking deficits. Participants were closely monitored over a period of 1–2 weeks, during which they underwent regular evaluations and training sessions. Four participants trained in the laboratory and were evaluated daily for 6 days (day 1 to day 6, spread over 2 weeks), while five participants trained for 6 days (in the lab on days 1 and 6, at home on days 2–5) and were evaluated in the laboratory on days 1 and 6. The primary outcome was gait speed (measured in the 10-m walk test). Surface electromyography (EMG; recorded using Trigno Avanti sensors [Delsys, Inc.]), and leg kinematics were recorded on each evaluation day while participants performed the 10-m walk test four times. EMG signals were recorded from rectus femoris (RF) and adductor magnus (AM). The EMG signals were digitized at 1926 Hz and bandpass filtered using a Butterworth filter (50–450 Hz, forward and backward). To minimize measurement bias, the evaluators performing the 10-m walk and kinematics testing did not work with the participants during MINT conditioning. We chose a broad range of gait impairment to attempt to minimize selection bias.

### Training paradigm

Participants were asked to train with MINT for six days, 60 min per day. On average, participants were expected to complete ~ 30 repetitions per run, so we anticipated they would perform ~ 1080 reps of total training. Training was split into six runs of 10 min each; each repetition denotes one attempt to move the cursor to a target, with a time limit of 10 s. After completing each run, the participants were instructed to sit down and rest. MINT consists of a gaming rehabilitation system comprised of software and hardware [29]. The hardware includes a customized, wireless surface EMG system (Myomo, Inc) that amplifies, digitizes, and computes the EMG envelopes and transmits them via Bluetooth.

Each session began by performing a maximum voluntary contraction (MVC) before MINT conditioning started to calibrate the conditioning to each participant’s residual strength. Participants were instructed to maximize the activation of either RF or AM muscles in the toe-off position. The MVC and resting baseline values were used to personalize the mapping of EMG envelope to cursor movements for each participant.

**Fig. 1:**
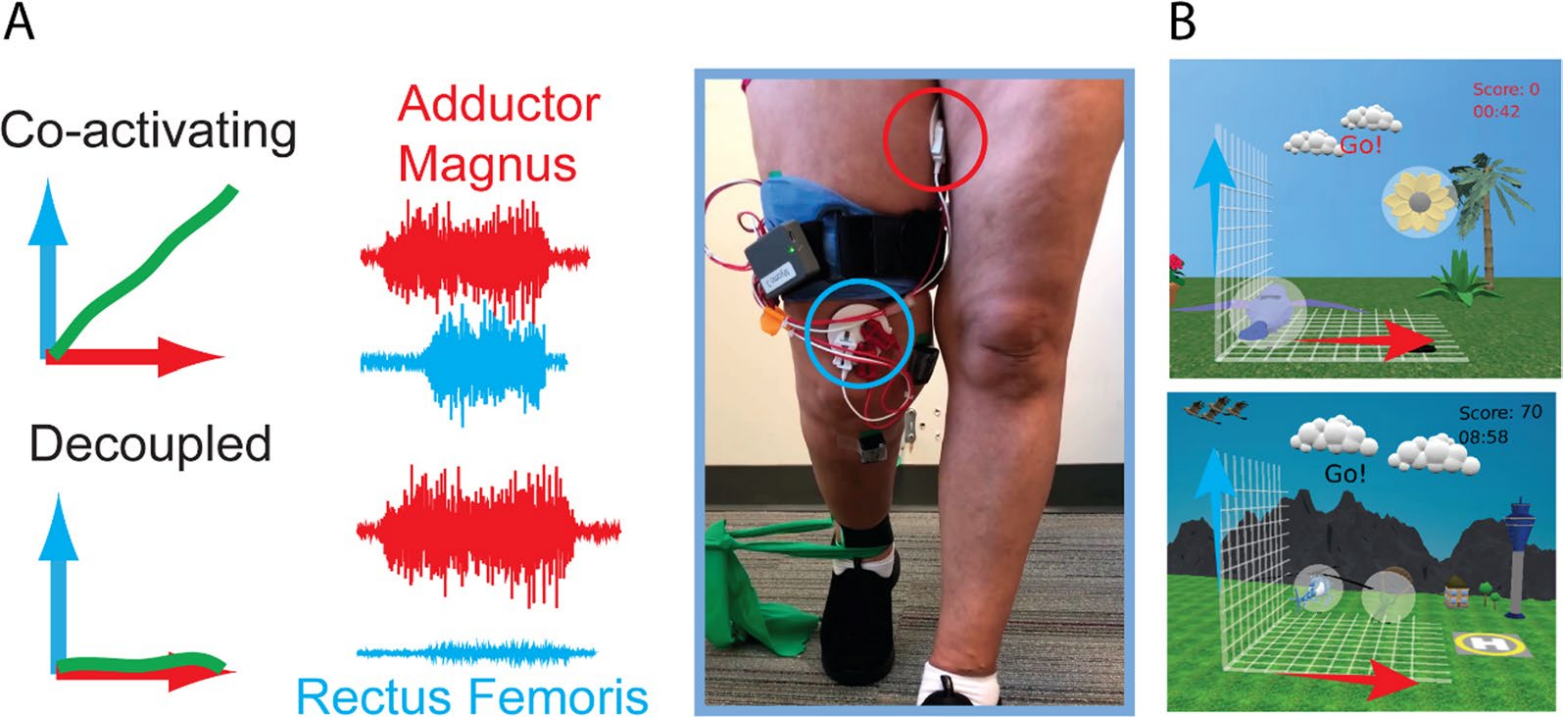
MINT paradigm. **A** Participant engaged in MINT conditioning using the wearable device. EMG signals from AM (red) and RF (blue) were mapped in orthogonal directions and vector summed to control the cursor's movement. When muscles were co-activated, the cursor moved along a diagonal between the two directions. To encourage the participant to separate the muscle activations, targets were gradually moved progressively further away from the diagonal until they were only in the "up" or "right" positions. **B** Various game skins were implemented based on participant preference to enhance enjoyment and engagement

During MINT conditioning, EMG envelopes from AM and RF were mapped to orthogonal components of cursor movement, and the cursor moved as a vector sum of the two components [29] (Fig. 1). After holding the cursor in the home target (at bottom left of the screen) by relaxing both muscles, an outer target appeared toward the opposite end of the screen, and participants attempted to move the cursor into that target and hold for 0.5 s. To enhance shaping and increase participant engagement the difficulty was gradually incremented across five key factors in the following order: increasing the angular separation between the outer target and the diagonal, decreasing cursor size, decreasing target size, increasing the requisite leg muscle relaxation after each trial, and increasing the muscle activation needed to acquire the outer target. Outer targets were initially placed at a distance equivalent to 15% of the MVC from the home target. The placement of outer targets occurred at random angles within a pre-defined range, originating in proximity to the 45° diagonal between muscles (signifying high co-activation) and progressing farther away from the diagonal as the difficulty level advanced. This progression mandated enhanced muscle isolation as the difficulty level increased.

Seven of the participants used MINT in a standing position with toe-off. The participants used a custom-designed 45° inclined foot brace to keep their foot in the toe-off position. Two participants used MINT in a sitting position due to being easily fatigued, although they were asked to keep their foot in the toe-off position using the foot brace. We taught home group participants how to use the MINT system and place electrodes in the laboratory, and they received daily technical support (if needed) via phone or video chat from lab staff. Automated algorithms were used to monitor MVC estimates and gameplay statistics daily and alert lab staff if issues arose.

### Outcome measures

Game performance metrics (success rate, time to target) were recorded for each 10-min game run. These performance metrics were monitored remotely by lab staff using a secure cloud server to ensure participant adherence. A lab member not involved in training performed functional evaluations of the 10-m walk test (primary outcome). Participants rested for at least 20 s between walks to avoid fatigue. The baseline co-activation between adductor magnus and rectus femoris was defined as the correlation coefficient between their EMG envelopes during the 10-m walk test. Leg kinematics during gait were measured using inertial measurement unit (IMU) sensors to examine effects on hip abduction and knee flexion angles. To determine the knee angle, we positioned two IMU sensors: one over the lateral epicondyle, and the other over the tibial tuberosity. For the hip angle, an IMU sensor was placed over the anterior superior iliac spine (ASIS). Using the IMUs and the measured limb lengths to create a kinematic chain model [31] representing the lower extremity, we calculated the 3D orientation of the leg and estimated the knee flexion angle. The IMUs over the ASIS and lateral epicondyle were used similarly to estimate the hip abduction angle.

### Safety

To monitor adverse events, we instructed our participants to report any incidents, such as falls while using MINT or any instances of pain or fatigue during its use. Participants were instructed to perform the MINT conditioning with a walker that was provided to them. Additionally, we advised participants to take a brief rest after each run.

### Statistical analysis

Paired t-tests were utilized to assess the significance of changes in gait speed and kinematic outcomes, including knee flexion angle and hip circumduction, following MINT conditioning. Unpaired t-tests were used to compare the number of repetitions between limited community ambulators and full community ambulators, as well as between responder and non-responder groups. Furthermore, one-way ANOVA was used to evaluate differences in muscle co-activation between learning curves over the 6-day period between limited and full-community ambulators and between responders (those who improved by at least the MCID) and non-responders (those who did not improve by at least the MCID). With one-way ANOVA we assessed the potential differences in co-activation between the two groups across the entire 6-day period, specifically without accounting for the influence of time. All statistical analyses were conducted using MATLAB with a significance level of p < 0.05 indicating statistical significance.

We did not encounter any missing values for either clinical or kinematic outcomes. However, there was a single missing value for the game performance on day 5 in one subject (out of 9 subjects, with data points collected over a 6-day period). To address this, the missing data point was replaced with the preceding day's recorded value of 4. This decision was made in accordance with the last observation carried forward (LOCF) method, a standard practice in cohort studies for managing missing data points [32].

## Results

**Fig. 2:**
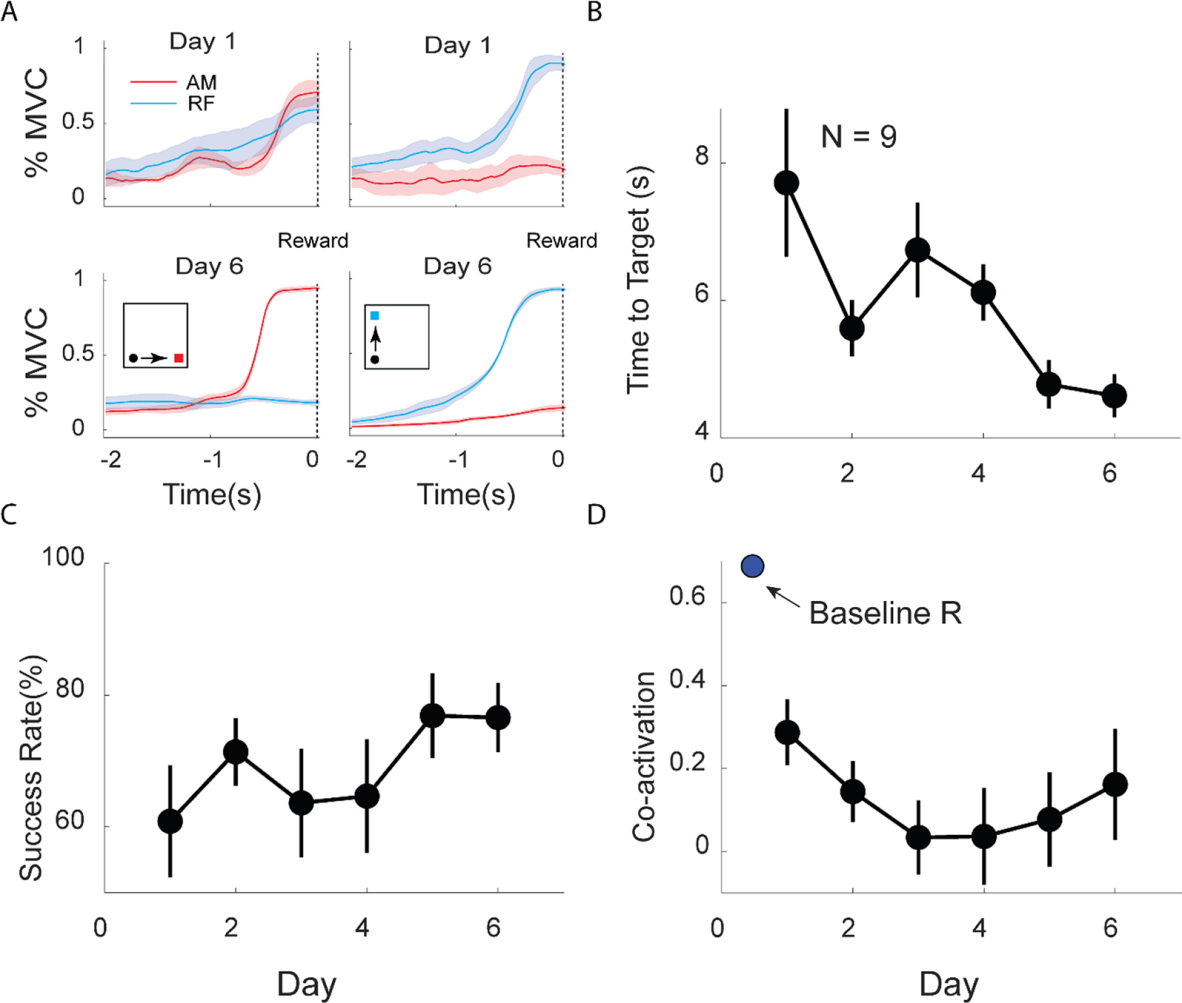
MINT conditioning improved game performance and decreased muscle co-activation. **A** Mean (± SEM) normalized EMG envelope in the 2 s before successful target capture (“Reward”) for all runs in days 1 (top) and 6 (bottom) for subject 1. Left plots show AM targets, right plots show RF targets (shown in insets). This participant learned to reduce activity in the non-targeted muscle by day 6. **B**, **C** Time-to-target and success rate (mean ± SEM) over participants improved over the course of MINT conditioning. **D** Mean co-activation decreased during conditioning, especially from baseline co-activation obtained during walking

Nine participants (4 women, 5 men, aged 60 ± 7 (mean ± SD) years) enrolled in this study. The mean time from stroke onset at enrollment was 10 ± 6 years. Strokes were located in the right hemisphere in 5, left hemisphere in 4. Of the total participants, 2 individuals identified as Hispanic, while the remaining 7 participants were non-Hispanic. During MINT conditioning, participants were operantly conditioned to reduce co-activation between adductor magnus and rectus femoris (Fig. 2A). Participants improved their MINT performance (increase in success rate and decreased time-to-target) over the 6 days (Fig. 2B, C). Participants reduced co-activation during MINT training between adductor magnus and rectus femoris by a mean of 70% compared to baseline (Fig. 2D). In total, participants completed 952 ± 287 repetitions over 6 days of training. The at-home group completed 970 ± 225 repetitions, while the in-lab group completed 938 ± 355 repetitions.

**Fig. 3:**
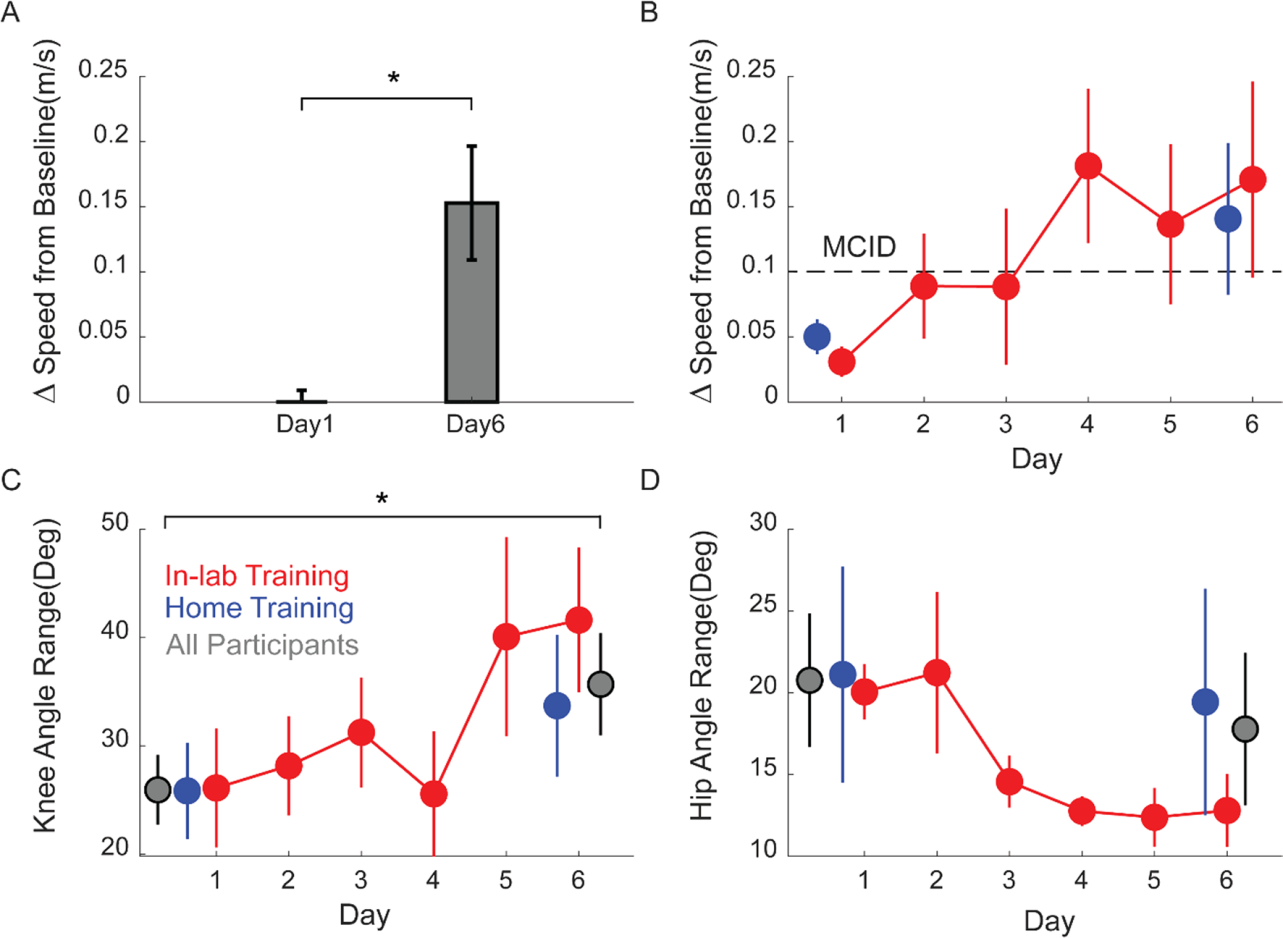
Functional outcomes of MINT conditioning. **A** Gait speed significantly improved by a (mean ± SE) of 0.15 ± 0.04 m/s across all participants, more than the MCID. **B** Both in-lab and home training led to improvements in walking speed. Each point represents the speed change from day 1 pre-training baseline sessions. **C** Knee flexion increased by 13° and **D** hip abduction showed a decreasing trend by 7° from baseline over all participants (gray). Each point shows the after-MINT training knee or hip angle. (* indicates statistical significance with p < 0.05)

Participants did not report any adverse events from MINT conditioning. Further, participants’ gait speed on the 10-m walk test increased by 0.15 m/s (p = 0.006, paired t-test) from day 1 baseline (prior to training) to after training on day 6 (Fig. 3A). This value was higher than the minimum clinically important difference (MCID) of 0.1 m/s. Both participant groups (those that trained at home and in lab) improved walking speed after training (Fig. 3B). In addition, while walking, participants’ knee flexion angle significantly increased 13° from pre-training day 1 baseline (p = 0.03, paired t-test) and hip abduction (circumduction) showed a non-significant decreasing trend (p = 0.24, paired t-test) of 7° from pre-training day 1 baseline (Fig. 3C, D). In-lab participants improved knee flexion and hip circumduction by 21° and 7° compared to pre-training day 1 baseline, respectively, while at-home participants improved knee flexion and hip circumduction by 10° and 6°, respectively. These differences between in-lab and at-home groups were not statistically significant (p = 0.4 and 0.9 for knee flexion and hip circumduction, respectively, unpaired t-test).

**Fig. 4:**
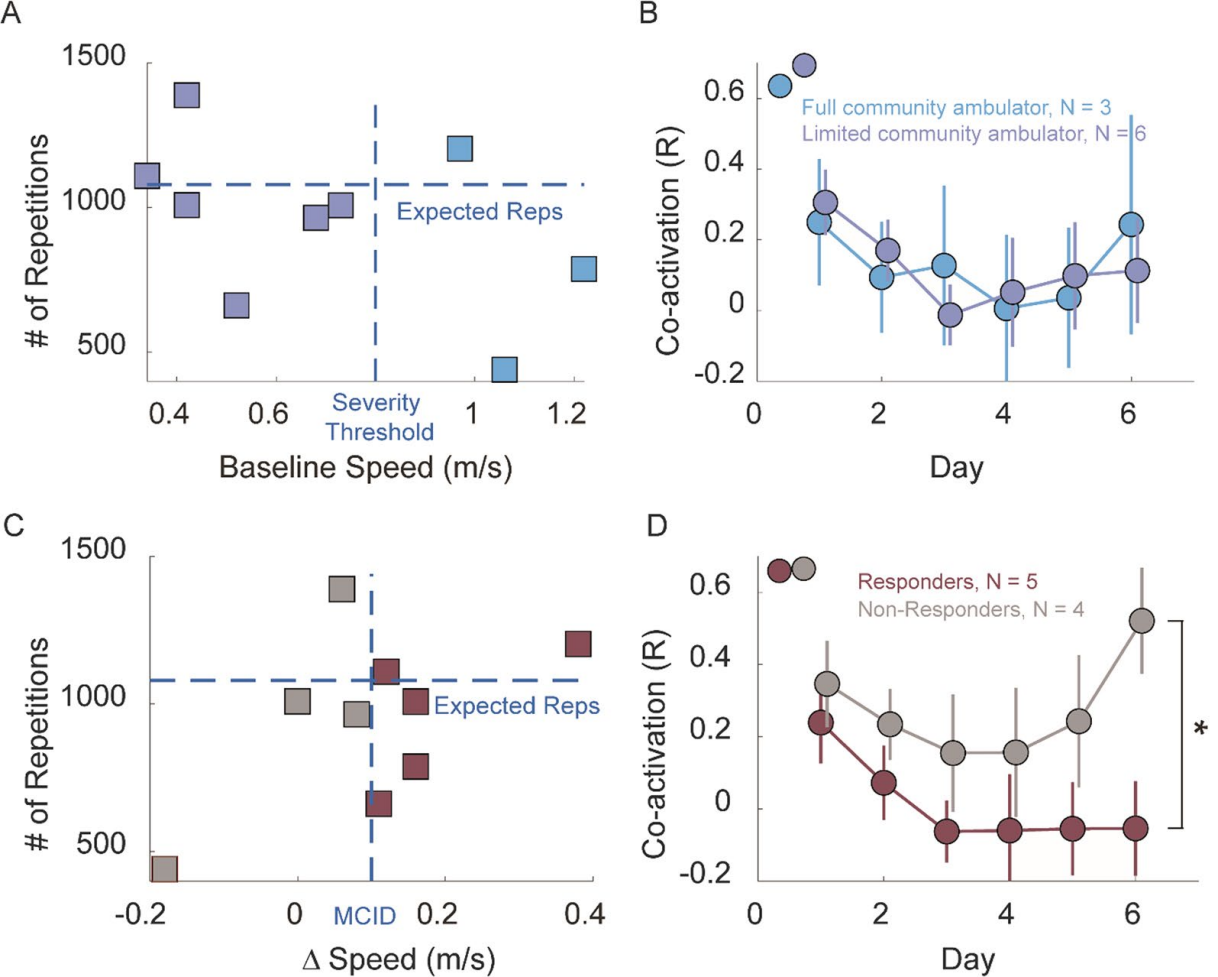
Effects of impairment severity and responder status on repetitions and abnormal co-activation. **A** Total number of repetitions vs. baseline speed for each participant (square). Horizontal dashed line represents the theoretical expected number of repetitions over 6 days and vertical dashed line divides participants into limited and full community ambulators. **B** Co-activation (R) between RF and AM during 6-day MINT conditioning for full and limited community ambulators. **C** Number of repetitions vs. gait speed change (day 1 to day 6) due to training. Horizontal dashed line is the same as in A; vertical dashed line shows MCID of 0.1 m/s used to divide participants to responders and non-responders. **D** Co-activation between RF and AM for responder and non-responder groups. (* indicates statistical significance with p < 0.05)

We analyzed whether stroke participants with more severe impairment engaged less in using the MINT device in terms of the number of repetitions, and if the severity of impairment affected the amount of repetitions that participants completed. We sorted the participants into two groups based on their walking speed: limited community ambulators with a gait speed of 0.4 to 0.8 m/s (n = 6), and full community ambulators with a gait speed between 0.8 and 1.2 m/s (n = 3) [33]. Full community ambulators completed (810 ± 380) repetitions over the 6-day period, while limited community ambulators completed (1023 ± 233) repetitions (Fig. 4A, p = 0.32, unpaired t-test). Additionally, there was no significant difference observed in the co-activation between the RF and AM muscles in the two groups during training (Fig. 4B, p = 0.95, one-way ANOVA).

To investigate whether training intensity influenced functional improvement, we divided the participants into responders and non-responders. Responders completed 954 ± 225 total repetitions over 6 days, while non-responders completed 950 ± 390 repetitions (Fig. 4C, p = 0.68, unpaired t-test). Figure 4D compares co-activation during training for responders and non-responders. Responders had a greater reduction in co-activation throughout the 6-day training. Notably, there was a significant difference observed in the RF/AM co-activation during training between responder and non-responder participants (p = 0.0022, one-way ANOVA). Moreover, responders increased their knee flexion angle by a mean of 23° from pre-training day 1 baseline, while non-responders increased this angle by only 2° (p = 0.007 between groups, unpaired t-test). Additionally, the hip circumduction angle in responders decreased by 10° in responders, compared to only 1° in non-responders (p = 0.6 between groups, unpaired t-test).

## Discussion

In this study, we investigated the safety, feasibility, and impact of MINT conditioning on abnormal co-activation between AM and RF and walking function in chronic stroke survivors. We tested the hypothesis that reducing abnormal hip adduction/knee extension co-activation would improve walking function. Participants trained with MINT safely, with no adverse events even when training at home while standing. MINT conditioning is feasible—as evidenced by a high number of repetitions, improved performance, and reduced co-activation—both in the lab and at home. After just six days of MINT conditioning, participants improved walking function significantly and by more than the MCID. This was true of both in-lab and at-home use of MINT. Gait biomechanics improved as well. This preliminary causal evidence suggests that abnormal co-activation between hip adductors and knee extensors does indeed contribute to gait dysfunction after stroke [12] and suggests that reducing it could improve walking function.

To the best of our knowledge, there are no previous rehabilitation studies designed specifically to counteract abnormal co-activation in the leg after stroke. In particular, there exist none that address hip adductor-knee extensor co-activation. The results here, though uncontrolled, suggest that therapies addressing this issue may help walking function, as well as arm function. They further suggest that MINT conditioning warrants further study of efficacy in a longer, larger, randomized controlled trial. MINT can provide an enjoyable, and ultimately affordable, game-based solution for at-home rehabilitation, which encourages participants to engage in high doses of training at home. Its new mechanism of action reduces abnormal co-activation, which is not typically addressed in conventional therapies. The ability to train at home is advantageous, as it could enable higher dosage and greater penetration into underserved communities.

Importantly, MINT was used as much for stroke survivors with limited community ambulation as those with community ambulation. The innovation of MINT conditioning lies in providing a wearable (and ultimately affordable) rehabilitation option that specifically targets abnormal co-activation in the leg. All participants demonstrated a high level of engagement, and limited community ambulators performed the expected number of repetitions over the six-day period. This finding suggests that MINT conditioning was motivating for more severely impaired individuals. Further, even limited community ambulators participants could learn to reduce abnormal co-activation (Fig. 4B). This aligns with motor learning studies indicating that unilateral stroke does not impair the acquisition of motor skills [34, 35]. It also aligns with our prior MINT conditioning studies in the arm [28, 29], in which even those with severe arm impairments could use and benefit from MINT. This population, often excluded from clinical trials, typically is most in need of new therapies.

We also investigated the relationship of training dose and responder status with co-activation. Both responders and non-responders achieved a high number of repetitions, with no significant difference between them. In contrast, the co-activation curve of responders remained significantly lower than non-responders over all days, in particular days 3–6 (Fig. 4D). This suggests that learning to reduce abnormal co-activation was an important factor in explaining the improvement observed in responders. The rapid change in the learning curve (days 1–2) supports the notion that participants quickly adapted to using MINT to decouple these muscles (Fig. 2D). Moreover, the responder group exhibited greater improvement in joint biomechanics than did non-responders, specifically a greater increase in knee flexion and trend of greater reduction in abnormal hip circumduction. This suggests that these improvements in function from MINT conditioning are a result of improved biomechanics, rather than some other compensatory mechanism. It further suggests that the abnormal coupling of hip adductors with knee extensors does indeed contribute significantly to impaired joint kinematics.

It is not clear what specifically causes abnormal hip adductor/knee extensor co-activation. In the arm, it has been proposed that abnormal co-activation results from reduced availability of the corticospinal tract (CST), leading to a compensatory reliance on other tracts, particularly the corticoreticulospinal tract. While this may be the cause for abnormal hip adductor/knee extensor co-activation as well, some findings also indicate that could be attributed to changes in the polysynaptic spinal reflexes [36]. In addition to this abnormal co-activation pattern, others have been reported [12, 23]. Thus, it is possible that MINT conditioning could help other abnormal co-activation patterns in the leg as well. Although there is substantial evidence suggesting that MINT improves movement by changing the co-activation patterns of only the targeted muscles [27], the specific locations of plastic changes in the brain or spine from this training remain unclear and are a subject for future investigations.

Our study had some limitations. While the number of participants was relatively small, we were still able to observe significant improvements in both walking function and knee kinematics. Without a control group, we cannot definitively attribute the observed improvements to the MINT intervention and rule out the possibility that any type of training could lead to similar outcomes. Nevertheless, the fact that responders were able to decouple abnormal hip adductor and knee extensor muscles to a greater extent than non-responders support the likelihood that MINT conditioning’s ability to reduce co-activation is important for improving arm function. The fact that significant effects were seen after just 6 days of training was remarkable and encouraging. The optimal dose remains to be determined. Finally, the current design of the MINT device may pose challenges for users. Its cumbersome nature could potentially limit its usability and acceptance among stroke survivors. Addressing this issue and developing a more user-friendly design is important for future iterations of the device. Despite these limitations, our study provides valuable insights into the potential benefits of MINT for improving walking function in stroke survivors with abnormal co-activation. We plan to investigate MINT conditioning more closely in a randomized, controlled trial of longer duration that will inform us about effects of training duration as well as the potential for sustained improvement of leg function.

## Conclusion

This study suggests that MINT conditioning led to a significant reduction in abnormal co-activation during training and improved walking function and kinematics. This suggests that abnormal co-activation contributes to gait impairment after stroke, and that reducing this co-activation may improve function. Overall, our study contributes to the understanding of gait dysfunction after stroke and highlights the potential of MINT conditioning as a wearable approach to improve walking function in stroke survivors.

## Data Availability

The data used in this study may be made available by the corresponding author upon a reasonable request.

## Abbreviations

EMG: Electromyogram
MINT: Myoelectric interface for neurorehabilitation
VL: Vastus lateralis
RF: Rectus femoris
VS: Vastus medialis
AM: Adductor magnus
BF: Biceps femoris
TFL: Tensor fasciae latae
MVC: Maximum voluntary contraction
IMU: Inertial measurement unit
ASIS: Anterior superior iliac spine
MCID: Minimum clinically important difference
